# Peripheral Giant Cell Granuloma: A Review of 123 Cases

**Published:** 2009

**Authors:** Niloofar Shadman, Shahram Farzin Ebrahimi, Shahin Jafari, Mohammad Eslami

**Affiliations:** * Assistant professor, Department of Restorative Dentistry, School of Dentistry, Kerman University of Medical Sciences, Kerman, Iran; ** Associate professor, Department of Oral Medicine, School of Dentistry, Tehran University of Medical Sciences, Tehran, Iran; *** Professor, Department of Oral Pathology, School of Dentistry, Tehran University of Medical Sciences, Tehran, Iran

**Keywords:** Giant cell epulis, giant cell granuloma, trauma

## Abstract

**Background:**

Peripheral giant cell granuloma is one of the reactive hyperplastic lesions of the oral cavity, which originates from the periosteum or periodontal membrane following local irritation or chronic trauma. The purpose of this study was to present the clinical characteristics of peripheral giant cell granuloma in a group of Iranian population.

**Methods:**

A series of 123 consecutive confirmed cases of peripheral giant cell granuloma after biopsy were evaluated. Age, sex, anatomic location, consistency, etiologic factor, pain and bleeding history, color, surface texture, and pedicle situation were recorded and were analyzed by chi-square test and values were considered to be significant if P < 0.05.

**Results:**

Age ranged from 6 to 75 years (mean 33 years). Women affected more than men (M/F 1:1.1). Peripheral giant cell granuloma was seen in the mandible more than in the maxilla and in the anterior region more than in the posterior region. In most cases, lesions were pink, pedunculated and had non-ulcerated surface. In less than half of the cases, there was no history of bleeding and also pain was rarely reported. Calculus was the most common etiologic factor.

**Conclusion:**

The results confirmed that the clinical features of peripheral giant cell granuloma in a group of Iranian population are almost similar to those reported by other investigators.

## Introduction

Chronic trauma can induce inflammation, produce granulation tissue with endothelial cells, chronic inflammatory cells and fibroblasts proliferation and manifests as an overgrowth called reactive hyperplasia.^1^ These tumor-like lesions are not neo-plastic, but they indicate a chronic process in which an exaggerated repair occurs (granulation tissue and formation of scars) following injury.^2^,^3^

**Figure 1:**
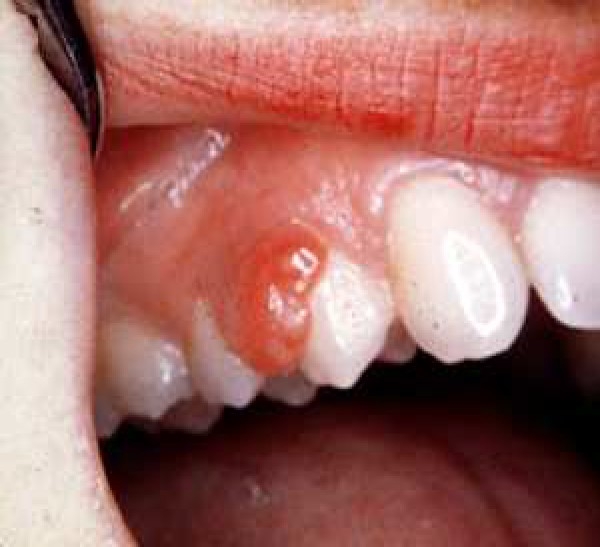
Initial appearance of peripheral giant cell granuloma

Reactive hyperplastic lesions are categorized to several groups.^1^ Peripheral giant cell granuloma (PGCG) is one of the most frequent giant cell lesions of the jaws and originates from the connective tissue of the periosteum or the periodontal membrane.^4^ It is not a true neoplasm but rather a benign hyperplastic reactive lesion occurred^4^ in response to local irritation such as tooth extraction, poor dental restorations, ill-fitting dentures, plaque, calculus, food impaction and chronic trauma (Figure 1).^5^ Other names of this lesion are peripheral giant cell tumor, osteoclastoma, reparative giant cell granuloma, giant cell epulis and giant cell hyperplasia of the oral mucosa.^4^

**Figure 2:**
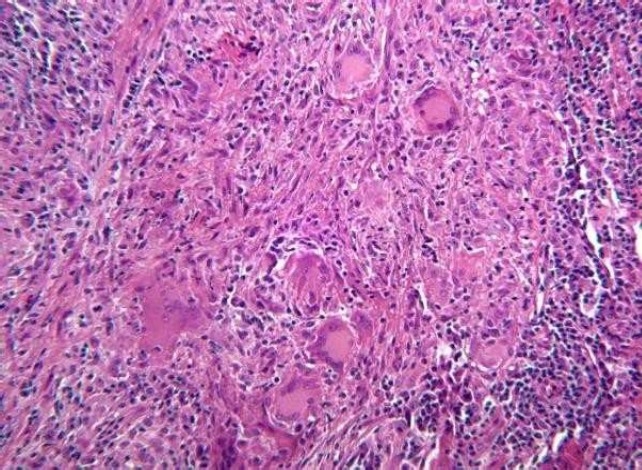
Histological appearance of peripheral giant cell granuloma. Fibrillar and reticular connective tissue stroma with abundant young connective tissue cells of fusiform shape, and multinucleated giant cells (H&E stain, magnification X400).

Histologically, PGCG is described as a non-encapsulated mass of tissue, containing numerous multinucleate osteoclast-like giant cells lying in a very cellular and vascular stroma (Figure 2).^6^

Clinically, PGCG manifests as a firm, soft, bright nodule or as a sessile or pedunculated mass and with occasionally ulcerated surface. The color, ranges from dark red to purple or blue.^4^ It is located in the interdental papilla, edentulous alveolar margin or at the marginal gingival level.^4^,^5^,^7^,^8^

They vary in size, though are rarely reported to exceed 2 cm in diameter.^9^ However, there have been reports of masses in excess of 5 cm, where factors such as deficient oral hygiene or xerostomia appear to play an important role in lesion growth.^4^ Incipient lesions may bleed and induce minor changes in gingival contour but large ones adversely affect normal oral function.^4^ Pain is not a common characteristic, unless they interfere with occlusion, in which case they may ulcerate and become infected.^9^ In some cases the underlying bone, suffers erosion and cup-shaped radiolucency occurs.^1^ The lesion can develop at any age. It is, however, more common in the fifth and sixth decades of life with a slight female predilection.^5^

Treatment comprises surgical resection, with extensive clearing of the base of the lesion to avoid relapse.^5^ The characteristics and clinical behavior of PGCG may vary in different populations and be difficult to predict, reflecting different environmental influences, lifestyles, and racial factors, assessment of which may help in the diagnosis and management. Information regarding gender, age, signs, and symptoms may be useful and lead to an early diagnosis and proper management, preventing further damage to hard and soft tissues of involved areas.

The purpose of this study was to describe the clinical features of 123 cases of PGCG at Tehran School of Dentistry, during a 4-year period

## Materials and Methods

The objective of this case series study was determination of clinical characteristics of all cases of PGCG that were biopsied and examined histologically at Tehran School of Dentistry between April 2001 and April 2005.

After preparing a medical chart on patients’ age and sex and characteristics of lesion such as anatomic location, color, consistency, characteristics of lesion base (pedunculated or sessile), patients with oral lesions with a PGCG like lesion (PGCG was one of the differential diagnosis of their oral lesions) were examined and charts were filled out. Then, for certain diagnosis and treatment, patients were referred to department of Oral Surgery for biopsy. After diagnosis of PGCG, the microscopic slides were reviewed by a second pathologist to confirm the histological diagnosis of PGCG.

The data collected by examining patients including age, sex and characteristics of lesions such as anatomic location, color, consistency, characteristics of lesion base (pedunculated or sessile), characteristics of surface, etiologic factor and a history of bleeding or pain were analyzed by chisquare test and values were considered to be significant if P < 0.05.

## Results

During the 4-year period, 123 cases of PGCG of oral cavity were biopsied and examined histologically at Tehran School of Dentistry.

### Age:

The mean age of the patients was 33 years (age range 6-75 years).

### Sex:

Out of 123 patients, 52.8% were females and 47.2% were males and the difference was not significant.

### Anatomic location:

PGCG was seen in the mandible (64.6%) more than in the maxilla (35.3%) and in the anterior region ”canine to canine region” (57.7%) more than in the posterior region “first premolar to back” (42.35%). The most common sites in descending order were posterior of the mandible (35.2%), anterior of the mandible (29.5%), anterior of the maxilla (28.2%), posterior of the maxilla (7.1%).

### Consistency and pedicle:

In 73.2% of cases, consistency was firm and in 61% of cases lesions were pedunculated.

### Surface characteristics, bleeding, color of lesion and pain:

Ulcerated surface was seen in 45% of cases, probably because of trauma. In 60% of cases, the tendency to bleed was noted. The color of lesions in most cases was pink. Purple, pinkred, and red colors were less frequent. In 96% of cases, there was no history of pain and in remaining cases, dull and slight pain was reported.

### Etiological factor:

In 40% of cases, no etiologic factor was found and in 31.8% of cases, calculus was the main etiologic factor. Other factors were inappropriate fillings, removable and fixed partial dentures and fracture in tooth structure.

## Discussion

Peripheral giant cell granuloma is a benign hyperplastic lesion caused by local trauma or chronic trauma. It originates from the periodontal ligament or mucoperiosteum.^4^

Tehran is the most crowded city in Iran with a population of over 7 millions and School of Dentistry at Tehran University of Medical Sciences is the main referral center for patients with various oral cavity diseases.

In this study, the age of patients ranged from 6 to 75 years with the mean age of 33 years and with the highest incidence in the fourth decade of life, similar to other studies mentioned below. Shafer and Levy,^10^ Giansanti and Waldron^11^ and Reichart and Philipsen^12^ reported the average age of 30 years. Katsikeris et al believed the peak incidence to be between 4^th^and 5^th^decades.^8^ Motamedi et al reported the average age of 31 years.^5^

PGCG affects females more than males,^2^,^7^,^8^,^11^ with a proportion of 1:1.5 or 1:2 according to Reichart and Philipsen^12^ or Giansanti and Waldron studies^11^ respectively. However Bhaskar et al,^13^ Salum et al,^14^ Zhang et al^15^ and also Murat et al^16^ reported a slight predilection for the male sex. But in some studies, PGCG had an equal prevalence in both genders.^3^,^5^ In our study, females were affected more than males, but the difference was not significant.

PGCG is more common in the lower jaw rather than the upper jaw.^5^,^11^,^17^ The reported proportion is 2.4:1 and in most cases, it occurs anterior to molar region.^1^,^7^,^11^,^12^ However, according to Pindburg^18^ the preferential location is the premolar and the molar zones. According to Motamedi et al, PGCG more frequently involves the mandible, commonly in the areas posterior to canines.^5^ In this study, lesions occurred more in the mandible and in the anterior region.

The consistency of lesions was dependent on the age of lesions because as time passes, maturation of lesions (increasing in collagen fibers) occurs and consistency shifts from soft to firm. In most studies, superiority in pedicle type was not mentioned.^1^,^2^,^19^ Regezi et al noted that PGCG was sessile,^3^ but in this study most lesions were pedunculated. Ulceration and bleeding can occur secondary to trauma.^3^ In this study more than one half of the cases had normal (non-ulcerated) surface. Pain was a rare finding in this study and it was not mentioned in similar studies either. Different etiologic factors were associated with PGCG, including complicated dental extractions, dental restorations in poor conditions, food impaction (dental apposition), plaque, calculus, etc.^8^,^20^,^21^ In this study, there was no etiologic factor in more than half of the cases, and calculus was the most causal factor.

On the other hand, Gunhan et al^22^ in their study on 26 PGCG cases suggested that these lesions could be influenced by sex hormones, and the giant cells were to be a potential target for estrogen (but not progesterone) action.^4^ In rare cases, giant cell granulomas are oral manifestations of hyperparathyroidism, when multiple lesions are identified and the patient suffers recurrences in spite of adequate treatment.^4^,^10^ The lesions typically associated with hyperparathyroidism appear centrally in bone and are referred to as brown tumors. In the lower jaw, these intrabony lesions can perforate the cortical layer, spreading towards the soft tissues and imitating a peripheral lesion.^4^ A parathyroid tumor or chronic renal failure primarily or secondarily can give rise to increased parathyroid hormone (PTH) production, which in turn favors the formation of giant cell lesions. Children with hypophosphatemic rickets (subclinical hyperparathyroidism) are also at an increased risk of developing such lesions. Histologically, brown tumors cannot be distinguished from giant cell granulomas.^4^

The treatment of PGCG comprises surgical resection with elimination of the entire base of the lesion and suppression of the etiologic factor. If resection is only superficial, the growth may recur. Most lesions respond satisfactorily to thorough surgical resection, with exposure of all the bone walls. When the periodontal membrane is affected, extraction of the adjacent teeth may prove necessary to insure full resection though this is initially contraindicated.^4^,^23^

In conclusion, the early and precise diagnosis of PGCG, allows conservative management with a less risk for teeth and adjacent bone.^4^
